# Testing the Feasibility of Remote Patient Monitoring in Prenatal Care Using a Mobile App and Connected Devices: A Prospective Observational Trial

**DOI:** 10.2196/resprot.6167

**Published:** 2016-11-18

**Authors:** Kathryn I Marko, Jill M Krapf, Andrew C Meltzer, Julia Oh, Nihar Ganju, Anjali G Martinez, Sheetal G Sheth, Nancy D Gaba

**Affiliations:** ^1^ George Washington University School of Medicine and Health Sciences Washington, DC United States; ^2^ OB Hospitalist Group Baylor All-Saints Medical Center Dallas, TX United States; ^3^ Center for Healthcare Innovation and Policy Research George Washington University School of Medicine and Health Sciences Washington, DC United States; ^4^ The Jackson Laboratory Farmington, CT United States

**Keywords:** prenatal care, pregnancy, mobile app, remote pateint monitoring

## Abstract

**Background:**

Excessive weight gain and elevated blood pressure are significant risk factors for adverse pregnancy outcomes such as gestational diabetes, premature birth, and preeclampsia. More effective strategies to facilitate adherence to gestational weight gain goals and monitor blood pressure may have a positive health benefit for pregnant women and their babies. The impact of utilizing a remote patient monitoring system to monitor blood pressure and weight gain as a component of prenatal care has not been previously assessed.

**Objective:**

The objective of this study is to determine the feasibility of monitoring patients remotely in prenatal care using a mobile phone app and connected digital devices.

**Methods:**

In this prospective observational study, 8 women with low risk pregnancy in the first trimester were recruited at an urban academic medical center. Participants received a mobile phone app with a connected digital weight scale and blood pressure cuff for at-home data collection for the duration of pregnancy. At-home data was assessed for abnormal values of blood pressure or weight to generate clinical alerts to the patient and provider. As measures of the feasibility of the system, participants were studied for engagement with the app, accuracy of remote data, efficacy of alert system, and patient satisfaction.

**Results:**

Patient engagement with the mobile app averaged 5.5 times per week over the 6-month study period. Weight data collection and blood pressure data collection averaged 1.5 times and 1.1 times per week, respectively. At-home measurements of weight and blood pressure were highly accurate compared to in-office measurements. Automatic clinical alerts identified two episodes of abnormal weight gain with no false triggers. Patients demonstrated high satisfaction with the system.

**Conclusions:**

In this pilot study, we demonstrated that a system using a mobile phone app coupled to remote monitoring devices is feasible for prenatal care.

## Introduction

Excessive weight gain and elevated blood pressure are significant risk factors for adverse pregnancy outcomes such as gestational diabetes, premature birth, and preeclampsia [1-4]. The impact of utilizing a remote patient monitoring system to monitor blood pressure and weight gain as a component of prenatal care has not been previously assessed. Given current technology, tools to measure weight gain and blood pressure are generally affordable, readily available, and may be connected to mobile devices for data transfer to medical providers. In addition, the utilization of these technologies may promote self-care and improve overall engagement with prenatal care [5]. Mobile phone technology has been previously shown to improve disease management for diabetes self-care activities, HIV infection medication adherence, and sickle cell anemia medication adherence [6-8]. We hypothesize that using digital health tools (a mobile app and connected monitoring devices) may enhance prenatal care.

The purpose of this study is to determine the *feasibility* of using digital health tools to manage prenatal care. Feasibility was determined by studying the following specific outcomes: (1) patient engagement with the app and the remote monitoring tools, (2) accuracy of the remotely collected data, (3) efficacy of the alert systems, and (4) patient satisfaction.

## Methods

### Setting and Subject Selection

This prospective observational study was conducted between July 2014 and January 2015 in the Department of Obstetrics & Gynecology at the George Washington University Hospital, an urban academic medical center that delivers approximately 2900 babies per year. Pregnant women between the ages of 18 to 40 years old presenting for routine prenatal care in the first trimester were asked to participate in the study over the course of a recruitment period of one month. Inclusion criteria included self-reported regular usage of an iPhone and low-risk pregnancy status per established guidelines [9]. In total, 8 participants were enrolled in the study and were followed until their delivery.

There was no cost to the patients or provider for participation. Once consented, participants were given access to the Babyscripts (Washington, DC) mobile prenatal care platform consisting of a mobile phone app and connected devices. Patients received training on how to use the app and the devices as part of the enrollment process. All patients signed an end-user licensing agreement to use the app, permitting Babyscripts to access non-identifiable data collected by the app. Institutional review board (IRB) approval was granted prior to commencing the study (IRB# 051422).

### Description of the Digital Health Platform

**Figure 1:**
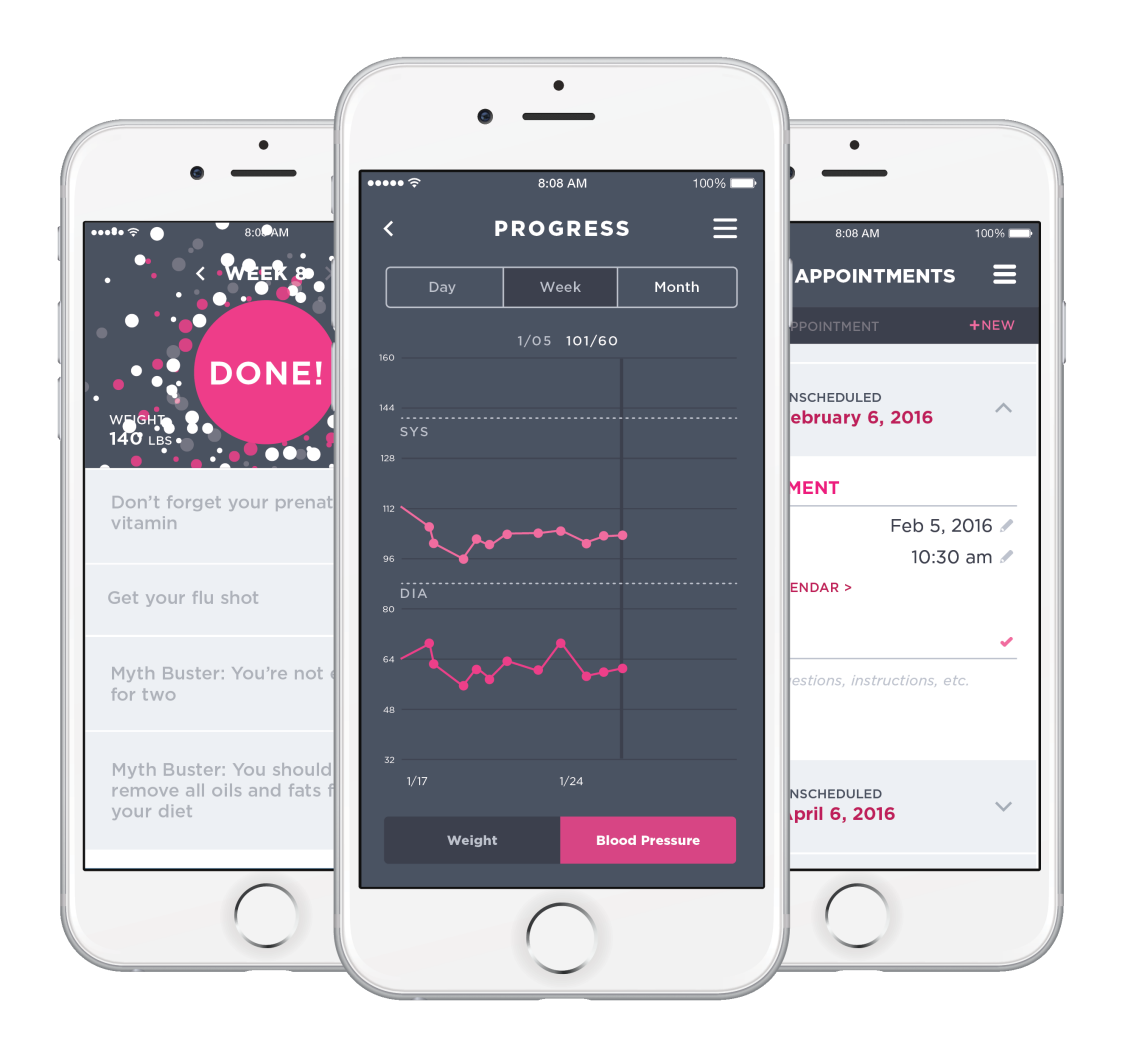
Screenshots of the Babyscripts app.

The Babyscripts platform was designed through a collaboration between the George Washington University Medical Faculty Associates and 1Eq Inc., the manufacturer of Babyscripts. The platform consists of a mobile phone app connected to a wireless weight scale and sphygmomanometer. The Babyscripts app contains evidence-based educational information related to prenatal care delivered at gestational age-specific times during pregnancy in the form of a to-do list (Figure 1). This information encompasses material covering pregnancy progression, modifiable risks such as alcohol intake, smoking or drug abuse, and information regarding nutrition, breastfeeding, appropriate weight gain, and pregnancy warning signs. This content was developed and validated in partnership with a committee of 3 board-certified obstetrician-gynecologists at the George Washington University Medical Faculty Associates. All items were derived from evidence-based standards supported by American Congress of Obstetrics and Gynecology (ACOG) and then further reviewed by each member of the committee.

### Connected Devices

In addition to the Babyscripts app, participants received connected devices including a weight scale (Smart Body Analyzer, Withings) and a sphygmomanometer (Wireless Blood Pressure Monitor, Withings). As part of participation, patients collected weight and blood pressure data on a weekly basis.

### Data Analysis and Alerts

Data points generated by the use of connected devices automatically populated the Babyscripts app for review by the patient as well as the provider. Patients were provided automated feedback about their individual weight and blood pressure goals. Abnormal values activated alerts to the patient and physician to communicate more urgently. For example, elevated blood pressure or abnormal weight gain or weight loss generated an automated alert to the clinician. The alert system consisted of an email to the office’s triage nurse as well as an email and an in-app notification to the patient. If the alert was not addressed in 15 minutes, an automated phone call was placed to the triage nurse to alert the provider of the abnormality. The alert remained active until it was acknowledged by the provider.

### Outcomes

#### Patient Engagement

Engagement with app and remote monitoring devices was measured by recording the number of times that a patient interacted with the app or recorded an at-home weight or blood pressure reading.

#### Accuracy of Remote Patient Monitoring

To measure the accuracy of remote monitoring, remote measurements were compared to in-office measurements. For in-office data, 2 trained abstractors reviewed the electronic medical record and then compared the data for discrepancies. Abstractors were not blinded to the study purpose. Standardized data collection sheets were used for data collection and all patient data was stored in a server compliant with the Health Insurance Portability and Accountability Act of 1996 (HIPAA).

#### Efficacy of Automatic Alerts

To measure the efficacy of the alert system, each patient’s home measurements were reviewed and abnormal values were cross-checked with the report of clinical alerts. If there were any discrepancies, a more detailed review was performed.

#### Patient Satisfaction

Patient satisfaction was measured using a 12-question survey that was completed by participants after 20 weeks of platform usage. Questions were based on established satisfaction surveys that measure patient-centered outcomes [10]. Survey creation was based on the Checklist for Reporting Results of Internet E-Surveys (CHERRIES) [11].

### Analysis

Statistical analysis was used to compare trends in the patient data collected remotely versus collected in-office. All statistical analyses were performed in the R programming environment (R Core Team, 2013) [12]. Standard statistical measures including *P* values and confidence intervals were calculated.

## Results

For this feasibility study, 8 patients were recruited and consented to participate at 8 to 10 weeks gestation, and were followed through delivery. Most patients were primiparous, married, with private insurance, and no major pregnancy risk factors. The age range of the participants was from 25 to 33 years with body mass indexes (BMIs) of 17.3 to 33.8 (Table 1). One patient (#313) was identified to have fetal intrauterine growth restriction, while another (#278) was identified to have preeclampsia during labor. Of the patients, 5 delivered via normal spontaneous vaginal delivery, 2 required primary cesarean deliveries at term for non-reassuring fetal heart tracing and arrest of dilation, while 1 requested a repeat cesarean delivery.

**Table 1:** Demographics, history, and delivery outcomes of each patient in the study.

Characteristic	Patient number							
	265	271	273	274	275	278	313	323
Age, years	31	29	30	33	28	30	31	25
Gravida/para	1/000	1/0000	1/0000	3/1011	1/0000	1/0000	1/0000	2/1001
Race	Caucasian	Caucasian	Caucasian	African American/ Hispanic	African	Hispanic	Asian	African American
Education	Graduate	N/A	Graduate	N/A	Graduate	Graduate	Undergraduate	N/A
Marital status	Married	Married	Married	Married	Married	Married	Married	Single
Payer	Private	Private	Private	Private	Private	Private	Private	Private
Tobacco use	No	No	No	No	No	No	No	No
Alcohol use	Occasional	No	No	No	No	No	First trimester	No
BMI	17.3	23.5	25.9	28.0	20.6	33.8	23.0	24.0
Past medical history	None	Anxiety, ADD^a^	None	LEEP^b^ x3; cesarean deliveryx1, HSV^c^	Sickle cell trait	None	None	None
Pregnancy complications	None	None	Marginal previa resolved in second trimester	None	None	Preeclampsia at term	IUGR diagnosed in second trimester	None
Delivery	NSVD^d^ at 37.5 weeks	NSVD by induction of labor for macrosomia prevention at 40.6 weeks	Primary cesarean delivery for NRFHT^e^ in labor at 38 weeks	Elective repeat cesarean delivery at 40.1 weeks	NSVD at 41.4 weeks	Primary cesarean delivery for arrest of dilation at 39.2 weeks	NSVD at 39.6 weeks	NSVD at 36.6 weeks

^a^ADD: attention deficit disorder

^b^LEEP: loop electrosurgical excision procedure

^c^HSV: herpes simplex virus

^d^NSVD: normal spontaneous vaginal delivery

^e^NRFHT: non reassuring fetal heart tracing

**Figure 2:**
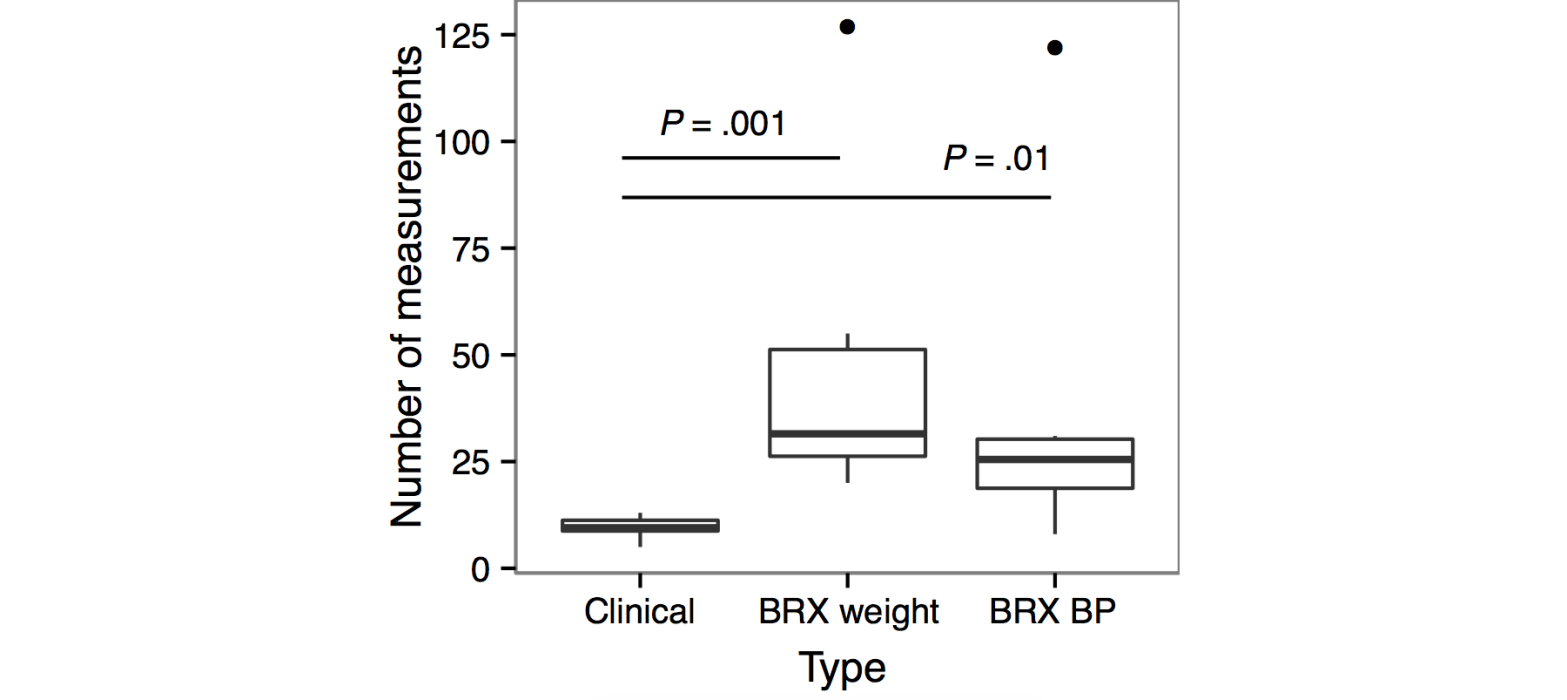
Comparison of average total number of measurements per individual over the course of pregnancy for patients with remote data collection versus in-office only. BRX: Babyscripts; BP: blood pressure.

**Figure 3:**
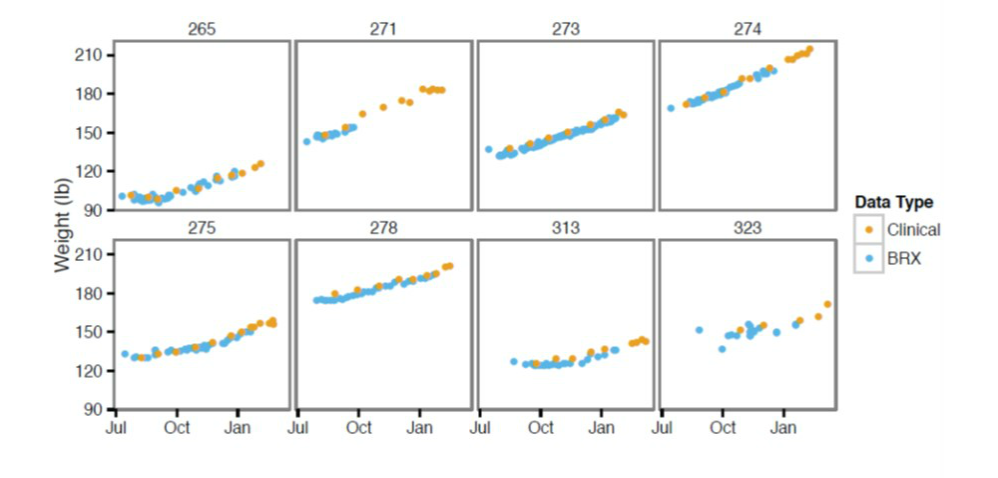
Comparison of weight values measured in office versus with remote digital device monitoring. Each box represents data points from an individual patient. BRX: Babyscripts.

**Figure 4:**
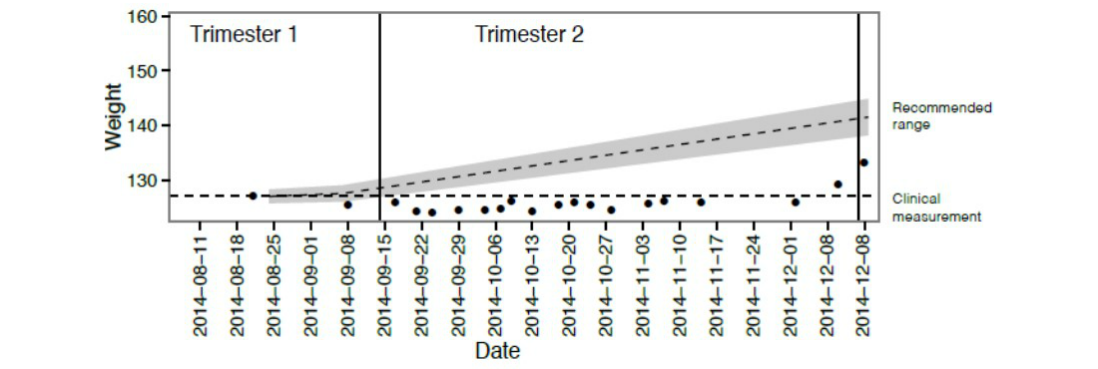
Weight gain for patient 313, who generated a clinical alert for poor weight gain.

**Figure 5:**
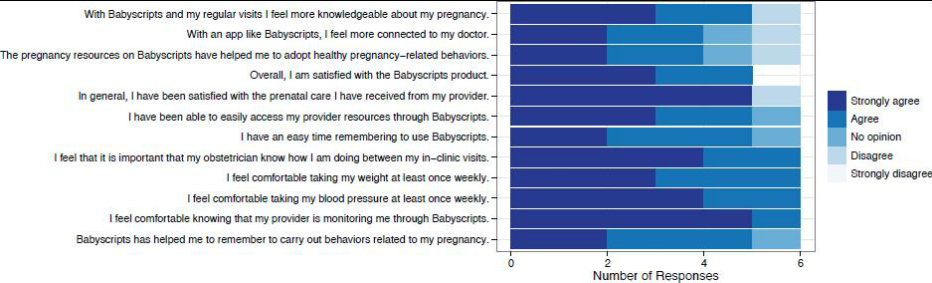
Distribution of survey responses for satisfaction with the Babyscripts experience.

### Patient Engagement

Patient interaction with the mobile app averaged 5.5 times per week over the 6-month study period. Weight data collection averaged 1.5 times per week and blood pressure data collection averaged 1.1 times per week. One patient (#323) stopped collecting data after 29 weeks gestational age due to residence change. Remote patient monitoring increased the total number of data points collected throughout pregnancy compared to routine office measurements during prenatal care visits (Figure 2). The mean number of weight measurements collected by the connected devices (46, *P*<.001) and mean number of blood pressure measurements (34, *P*=.01) exceeded the number of data points collected in the office (10).

### Accuracy of Remote Patient Monitoring

Weight measurements acquired by remote digital devices did not differ from in-office measurements (*P*>.05 for all patients) (Figure 3). The differences in the slope of gestational weight gain were determined by the F-statistic and resulting *P* values were Bonferroni-corrected. For all comparisons, adjusted is *P*>.05. For our cohort, mean blood pressure measurements remained consistent over the course of the pregnancy with a mild elevation (<10%) of in-office systolic blood pressure values compared to home measurement. Diastolic measurements tracked very closely with systolic measurements.

### Efficacy of Automatic Alerts and Alerts

After reviewing data sets of all clinical variables and all organized alerts, no incidences of inappropriate alerts or unaddressed alerts were discovered. There was a total of 2 alerts fired during the study, both related to inappropriate weight gain (patient 313 and 323). Patient 313 did not gain weight over a 4-week interval, which generated an automatic clinical alert. As a result of this alert, the patient’s obstetrician was notified and subsequently scheduled a more urgent office visit and closer monitoring (Figure 4). For patient 323, an automatic alert was generated at 15 weeks gestation for weight loss of 6 pounds in one week. The provider established contact with this patient and addressed any possible warning signs and monitored weight more closely until the patient was seen for her routine follow-up visit.

### Patient Satisfaction

The patient satisfaction survey assessed themes of patient-provider relationship, engagement, patient education, and patient satisfaction and had a completion rate of 75% (6/8) (Figure 5). We attempted multiple times to reach the 2 participants who did not complete the survey.

All 6 participants who completed the survey felt comfortable with the concept and technical aspects of remote monitoring, were able to easily access provider resources through Babyscripts, and had an easy time remembering to use Babyscripts. Most (83%, 5/6) of the participants felt that the app assisted with healthy pregnancy-related behaviors, were satisfied with prenatal care, felt more connected with their provider, and felt more knowledgeable about their pregnancy.

## Discussion

### Principal Findings

The main finding of this study was that the use of a novel pregnancy platform, which incorporates remote monitoring and a clinical alert system, is feasible as evidenced by high patient-app engagement, accurate at-home measurements of weight and blood pressure, efficacy of the alert system, and high patient satisfaction scores.

Interaction with the app met expectations and collection goals. The app was designed to provide regular educational information regarding pregnancy and prenatal care. Patient interaction with the mobile app averaged 5.5 times per week over the 6-month study period demonstrating that patients are visiting the app almost daily. The app was also designed to prompt patients to record weight and blood pressure at least weekly. Given these collection goals, the average interaction per week was chosen as a metric for engagement. Weight data collection averaged 1.5 times per week and blood pressure data collection averaged 1.1 times per week, which is a significant increase from the current standard of only recording blood pressure and weight during office visits. Based on discussions with experts, we concluded that more frequent readings were unlikely to add additional clinical information.

Accuracy of remote measurements are essential to make appropriate clinical management decisions. Remotely collected data tracked closely with in-office data demonstrating the accuracy of the remote devices. Blood pressure values measured in the office were mildly (<10%) elevated compared to the remote measurements, which has been previously described in the literature comparing at home blood pressure measurements to office measurements [13].

In addition, this platform demonstrated that automated alerts may be an effective way to notify the patient and provider regarding abnormal change in weight or blood pressure. The goal of the alert system is to facilitate earlier identification of pregnancy complications and optimize timely intervention. The described monitoring system has the ability to collect data more frequently than office visits alone, allowing for the potential to develop predictive models to screen normal pregnancies and identify pregnancy risk earlier.

### Limitations

The major limitations to this study include the small sample size and the threat of selection bias due to a convenience sample. It is possible that our results will not be reproduced in a different population or larger population. There are a number of possible biases within the participant population that limit our ability to demonstrate feasibility. The limitations based on the breadth of the study population include a mostly married cohort with private insurance, and 75% (6/8) patients were experiencing their first pregnancy. The fact that this was their first pregnancy makes it difficult for them to compare mobile prenatal care with other models of prenatal care. None of the women had significant past medical history nor used tobacco. Despite the attempt to choose a healthy cohort of women, 25% (2/8) of the women did experience a complication of pregnancy, specifically growth delay in one fetus and preeclampsia in another. While the sample size was small, it was ethnically diverse including 3 Caucasian women, 3 African-American women, 2 Hispanic women, and 1 Asian-American woman. Future studies will include examining the efficacy within specific ethnic and racial populations.

Second, researchers were not blinded to the purpose of the study, introducing the possibility of bias when assessing our primary outcomes. Finally, the lack of a comparison group limits the ability to draw conclusions about improved outcomes compared to usual prenatal care. However, this is a pilot study to determine the feasibility of a novel system, with plans for more rigorous studies in the future addressing these and other limitations.

### Conclusions

In the future, prenatal care is likely to incorporate more personalized care that integrates mobile technology, individualized risk stratification, and remote monitoring. As such, this study is the first to demonstrate the feasibility of using a digital health platform to remotely collect data in near real-time and to stratify for high-risk outcomes using an effective alert system. Future studies will compare prenatal care assisted by mobile health technology compared to routine care evaluating comparative-effectiveness and patient-centered outcomes.

## References

[1] Pattinson R, Say L, Souza JP, Van Den Broek N, Rooney C (2009). More effective strategies to facilitate adherence to gestational weight gain goals. WHO Maternal Death and Near-Miss Classifications. Bulletin of the World Health Organization.

[2] Creanga A, Berg C, Syverson C, Seed K, Bruce F, Callaghan W (2015). Pregnancy-related mortality in the United States, 2006-2010. Obstet Gynecol.

[3] Kiel D, Dodson E, Artal R, Boehmer T, Leet T (2007). Gestational weight gain and pregnancy outcomes in obese women: how much is enough?. Obstet Gynecol.

[4] The American Congress of Obstetricians and Gynecologists Hypertension and Pregnancy.

[5] Borrero AF, Vasques J, Vargas R (2015). Implementation of a mobile application to promote self-care in elder diabetic patients. VI Latin American Congress on Biomedical Engineering.

[6] Estepp J, Winter B, Johnson M, Smeltzer M, Howard S, Hankins J (2014). Improved hydroxyurea effect with the use of text messaging in children with sickle cell anemia. Pediatr Blood Cancer.

[7] Perera A, Thomas M, Moore J, Faasse K, Petrie K (2014). Effect of a smartphone application incorporating personalized health-related imagery on adherence to antiretroviral therapy: a randomized clinical trial. AIDS Patient Care STDS.

[8] Kim YJ, Rhee SY, Byun JK, Park SY, Hong SM, Chin SO, Chon S, Oh S, Woo J, Kim SW, Kim YS (2015). A smartphone application significantly improved diabetes self-care activities with high user satisfaction. Diabetes Metab J.

[9] The Management of Uncomplicated Pregnancy Working Group (2001). DoD/VA Clinical Practice Guideline for the Management of Uncomplicated Pregnancy.

[10] Hospital Consumer Assessment of Healthcare Providers and Systems HCAHPS Hospital Survey.

[11] Eysenbach G (2004). Improving the quality of Web surveys: the Checklist for Reporting Results of Internet E-Surveys (CHERRIES). J Med Internet Res.

[12] The R Project for Statistical Computing.

[13] http://hyper.ahajournals.org/cgi/content/long/62/6/982.

